# Population genetic diversity in an Iraqi population and gene flow across the Arabian Peninsula

**DOI:** 10.1038/s41598-020-72283-1

**Published:** 2020-09-17

**Authors:** Hayder Lazim, Eida Khalaf Almohammed, Sibte Hadi, Judith Smith

**Affiliations:** 1 Department of Biomedical and Forensic Sciences, College of Life and Natural Sciences, University of Derby, Derby, DE22 1GB UK; 2 Ministry of Interior of Qatar, Doha, Qatar; 3 School of Forensic and Applied Sciences, University of Central Lancashire, Preston, PR1 2HE UK

**Keywords:** Genetics, Haplotypes

## Abstract

Y-STRs have emerged as important forensic and population genetic markers for human identification and population differentiation studies. Therefore, population databases for these markers have been developed for almost all major populations around the world. The Iraqi population encompasses several ethnic groups that need to be genetically characterised and evaluated for possible substructures. Previous studies on the Iraqi population based on Y-STR markers were limited by a restricted number of markers. A larger database for Iraqi Arab population needed to be developed to help study and compare the population with other Middle Eastern populations. Twenty-three Y-STR loci included in the PowerPlex Y23 (Promega, Madison, WI, USA) were typed in 254 males from the Iraqi Arab population. Global and regional Y-STR analysis demonstrated regional genetic continuity among the populations of Iraq, the Arabian Peninsula and the Middle East. The Iraqi Arab haplotypes were used to allocate samples to their most likely haplogroups using Athey’s Haplogroup Predictor tool. Prediction indicated predominance (36.6%) of haplogroup J1 in Iraqi Arabs. The migration rate between other populations and the Iraqis was inferred using coalescence theory in the Migrate-n program. Y-STR data were used to test different out-of-Africa migration models as well as more recent migrations within the Arabian Peninsula. The migration models demonstrated that gene flow to Iraq began from East Africa, with the Levantine corridor the most probable passageway out of Africa. The data presented here will enrich our understanding of genetic diversity in the region and introduce a PowerPlex Y23 database to the forensic community.

## Introduction

The location of ancient Iraq corresponds to an area known as Mesopotamia^1,2^. This fertile land witnessed probably the first human settlement and cultural shift processes. It attracted the ancient hunter-gatherer people to settle down around 10,000 BC and initiate the agricultural society, which then developed to become a trading society^3^.

The Arabs were tribal people who inhabited the central Arabian Peninsula under the protection of many empires (Assyrian, Babylonian and others).

Modern Iraq is an Arabian country with a population of ~ 40 million, bordered by the Arabian Gulf, Kuwait, and Saudi Arabia to the south, Jordan and Syria to the west, Turkey to the north, and Iran to the east^4^. Supplementary Figure S1 shows the political borders of Iraq and its position in the Middle East^5^. There are five ethnic groups in Iraq but there is little published data about the diversity of the Iraqi population. In this context the major ethnic groups are Arabs and Kurds^6^. Our data represents the Arabs, the largest ethnic group.

SNP-markers are stable due to low mutation rates^7^; SNPs therefore have little diversity and weak discrimination for individual identification (unless used in large multiplexes). Therefore, in forensic practice, a combination of SNPs is used to determine haplogroups. This information also aids in studying human migration and evolutionary patterns^8^. In comparison, Y-STRs have an average mutation frequency of 0.2% per generation, with high levels of diversity and strong powers of discrimination between unrelated males, and can aid individual identification as well as our understanding of population structure and issues of consanguinity.

Recently, alleles at STR loci have been used to generate haplotypes^9,10^ and these haplotypes can then be used to predict a haplogroup and the population of origin^11,12^. Using this approach, Y-STRs can address internal diversity in the population by providing information on more recent events in the history of a haplogroup^13^. There is little published data about genetic diversity in the Iraqi population and its ethnic groups. This study utilises Y-STRs to shed light on the genetic makeup of this population, the relationship to its close neighbours and the effect of its colonisation history.

## Results

### Y-STR alleles and haplotype diversity within the Iraqi population

The PowerPlex Y23 loci showed more discriminating haplotypes than the Y-Filer kit. Supplementary Table S1 contains a full list of the Iraqi (Arab) haplotypes, as well as other sample information; data are also available from YHRD, release 62 (accession number YA004630).

Allele frequency distributions of the 23-STR loci and the most frequent allele for each locus are presented in Supplementary Table S2 for the 254 males of the population under study. Multiple alleles were observed for each locus ranging from 13 for DYS458 to four for DYS437. Genetic diversity and match probability values for each locus are presented in Supplementary Fig. S2 and Supplementary Table S3. By far the most polymorphic locus was DYS385, with a genetic diversity value of 0.93; the least polymorphic locus was DYS392 with a genetic diversity value of 0.34. The diversity of four of the six newly added markers for the PowerPlex Y23 kit (DYS481, DYS570, DYS576 and DYS643) showed greater diversity than the Y filer loci, as can be inferred from the ranking of these loci (ranks 3, 4, 5 and 7); the other two loci (DYS549 and DYS533) did not show such a high diversity and their ranks were 9 and 11 respectively.

Duplicated alleles were found in three Iraqi individuals at the locus DYS19. The three haplotypes show the same duplicated alleles (15, 16) and were predicted to belong to haplogroup G2a. These duplicated alleles were found in the same haplotypes that contain variant alleles at the locus (DYS385a/b). A null allele was found in two Iraqi samples at the locus DYS576 and these were predicted to belong to haplogroup J2.

The 254 Iraqi Arab males carried 244 distinct haplotypes, eight identical pairs, and one trio, providing a discrimination capacity of 96%. However, when the sub-set of Yfiler haplotype was considered, the shared haplotypes increased to 25, with a discrimination capacity of 85%. The summary statistics of diversity for PowerPlex Y-23 and Y-Filer kits for the 254 haplotypes of the Iraqi Arab population in this study are listed in Supplementary Table S4. The full list of haplotypes and their predicted haplogroups is presented in Supplementary Table S1.

### Microvariant alleles in the Arabian Peninsula

To study the microvariant alleles at the locus DYS458 in the Middle Eastern populations, the Middle Eastern data were compared to African, European, and southeast Asian countries. The presence of the microvariant alleles at the locus DYS458 was highest in the Middle Eastern populations (Table 1).

**Table 1:** Microvariant alleles at the locus DYS458 in different populations.

Population	Population size (n)	Number of microvariant alleles at the locus DYS458	Microvariant alleles (%) at the locus DYS458	References
Saudi Arabia	597	424	71	^14^
Yemen	128	80	62.5	*YA003764
Qatar	379	194	51.1	*YA004657
Kuwait	249	100	40.1	^15^
UAE	217	79	36.4	^16^
Iraq (Arabs)	254	88	34.6	This study
Lebanon	505	116	22.9	^17^
Egypt	208	43	20.6	^18^
Ethiopia	119	14	11.7	^19^
Iraq (Kurd)	104	12	11.3	^6^
Cyprus (Turkish)	253	17	6.7	^20^
Morocco	266	15	5.6	^21^
Eretria	161	7	4.3	^19^
Djibouti	54	2	3.7	^19^
Sweden	221	4	1.8	^17^
Belgium	207	3	1.4	^17^
Germany (Frieberg and Berlin)	391	3	0.7	^17^
India	256	0	0	^22^
South Africa	114	0	0	^17^
Kenya	228	0	0	^17,19^
South Korea	300	0	0	^17^
Finland	254	0	0	^17^
Japan (Gumma, Ibiraki, Tokyo)	259	0	0	^17^
China (Han), Chengdu (Han)	346	0	0	^17^

The presence of the microvariant alleles at the locus DYS458 was the highest in the Middle Eastern populations.

*YHRD accession number.

The total percentage of microvariant alleles in the Iraqi population was 36.6% (93/254). Most were observed at the locus DYS458 (88/254; 34.6%); 87 of these are predicted to belong to haplogroup J1, in particular the 0.2 variant which, was observed for alleles 17, 18, 19, 20 and 21. One individual with microvariant 15.1 was predicted to belong to haplogroup N. The rest of the microvariant alleles were distributed as follows: one copy of the duplicated STR DYS385a/b carrying a 0.2 variant (allele 13/14.2) was observed in four haplotypes and predicted to belong to haplogroup G2a. One haplotype carrying a 0.4 variant for allele 17 at locus DYS448 was predicted to belong to haplogroup J2a1b.

### Comparison with other populations

We compared the Iraqi population with other populations using Arlequin 3.5.2.2^23^ with the use of 10,000 permutations and 0.05 as the significance level. The population pairwise genetic distances (R_st_) were calculated between the Iraqi population and neighbouring Arab, Asian, African and European populations. The results are shown in Supplementary Table S5. The pairwise matric plot is shown in Supplementary Fig. S3.

The R_st_ pairwise differences were significant between the compared populations. The closest populations to the Iraqi Arabs were the Iraqi (Kurds) (R_st_ = 0.01081), then the Yemeni (R_st_ = 0.01215) and the Kuwaiti (R_st_ = 0.03986). The furthest were the Djiboutian (R_st_ = 0.24004), the Ethiopian (R_st_ = 0.22156) and the Turkish (R_st_ = 0.16422). Among the Middle Eastern populations Lebanon showed the highest genetic difference from the Iraqi Arabs (R_st_ = 0.14748).

The highest genetic difference was between Djiboutian and Iraqi (Kurds) (R_st_ = 0.25351) and the lowest was between Moroccan and Eritrean populations (R_st_ = 0.00714).

Arlequin 3.5.2.2 was also used to calculate the average pairwise differences between (PiXY) and within populations (PiX), in addition to the corrected average pairwise difference between populations (PiXY − (PiX + PiY)/2). The results are shown in Supplementary Table S6. The population average pairwise differences is shown in Supplementary Fig. S4.

Different groupings of Iraqis were compared with other populations and are shown in Supplementary Table S7. As expected, most of the variation occurs within populations, but variable values of the among-population variation were observed depending on the population groups targeted. This analysis suggested that Iraqis grouped best with Middle Eastern populations and all others as individual groups. The highest among-group difference was 3.52% and the lowest among-population within-groups variance was 6.75%; both of these values were noted when the Iraqi Arabs were grouped with the Middle Eastern populations. The P-values were significant for all among-group variance in various groupings.

Dendrogram clustering was illustrated based on R_st_ values using the R statistical software^24^, to display the relationships among the 23 populations; see Supplementary Fig. S5. Four clusters were created. Iraq (Arab), Iraq (Kurd), Yemen and Kuwait fell into one cluster. The rest of the Middle Eastern populations, including UAE, Qatar, Saudi Arabia, and Lebanon, fell into one cluster with Eritrea, Egypt, Morocco, South Korea and Japan. Three European countries, Sweden, Belgium and Finland, were clustered with China and India. The last cluster contained the three populations from African countries, Germany and Turkey.

The Iraqi and several Middle Eastern populations Y-STR data was analysed, using multi-dimensional scaling based on R_st_ distances using the R statistical software^24^. Supplementary Figure S6 shows the Multidimensional Scaling (MDS) plot of the Middle Eastern populations.

In the first dimension of the plot, four Middle Eastern populations lie in the lower left quadrant (Iraq (Arab), Iraq (Kurd), Yemen and Kuwait). Three European countries (Belgium, Finland and Sweden) are clustered with India and China in the upper left quadrant of the plot. All the other populations are clustered on the left side of the plot. The second dimension of the plot shows two clusters: the Middle Eastern occupies the lower two quadrants with some of the African countries and two Far Eastern countries (Japan and South Korea). All the European countries are clustered with some African and the South East Asian countries in the upper quadrants.

### Analysis of diversity via network analysis and haplogroup prediction

Whit Athey’s tool analysis showed that the Iraqi Arab population had seven major haplogroups; J1, E1b1b, J2a1b, J2, R1a, R1b and J2b. The most common haplogroup was J1 which represented 36.6% (93/254) of the population. The complete haplogroups for Iraqi Arabs are shown in Supplementary Table S8.

Median-joining Y-STR network was calculated for Iraqi Arab haplotypes with NETWORK v5.0.1.0. and edited using NETWORK Publisher v2.1.1.2 (Fluxus Technology Ltd)^25,26^. Based on Whit Athey’s Haplogroup Predictor, haplogroups were assigned to the Arab haplotypes within the network (see Supplementary Fig. S7).

The complete Iraqi Arab median-joining tree contains seven major clusters, each corresponding to a major haplogroup found in the Iraqi Arab population. All the predicted haplogroups form coherent clusters and create an accurate picture of the Y-STR dataset’s relation to the haplogroups. The most coherent clusters are J1, E1b1b and R1a, followed by J2, J2a, J2b and R1b which are the most spread-out.

### HapMap analysis for the Kidd Ancestry Informative SNPs (AISNPs) and the Y-STR data

Two HapMaps were generated using the program STRUCTURE which allows individuals to be clustered by their genetic information. The Kidd Ancestry Informative SNPs (AISNPs) using 55 SNPs from 140 populations (8,148 individuals)^27^ showed 10 clusters; and the HapMap of the Y-STR using 19 STR markers from 134 populations (21,323 individuals)^14–17^ showed 9 clusters.

The HapMap of the Kidd Ancestry Informative SNPs (AISNPs) showed an overlap between the North African and the South West Asian populations which include the Middle Eastern populations; and there was another overlap between the South West Asian and European populations. There was, however, poor sub-grouping of the countries within each population (see Supplementary Fig. S8). The HapMap of the Y-STR, the worldwide populations and the identified clusters of individuals corresponded to specific geographical regions without any overlap, with the Middle Eastern populations forming their own cluster. The HapMap of the Y-STR also showed a stronger sub-grouping of countries within each population (see Supplementary Fig. S9).

### Estimation of migration rate in the Iraqi population

**Figure 1:**
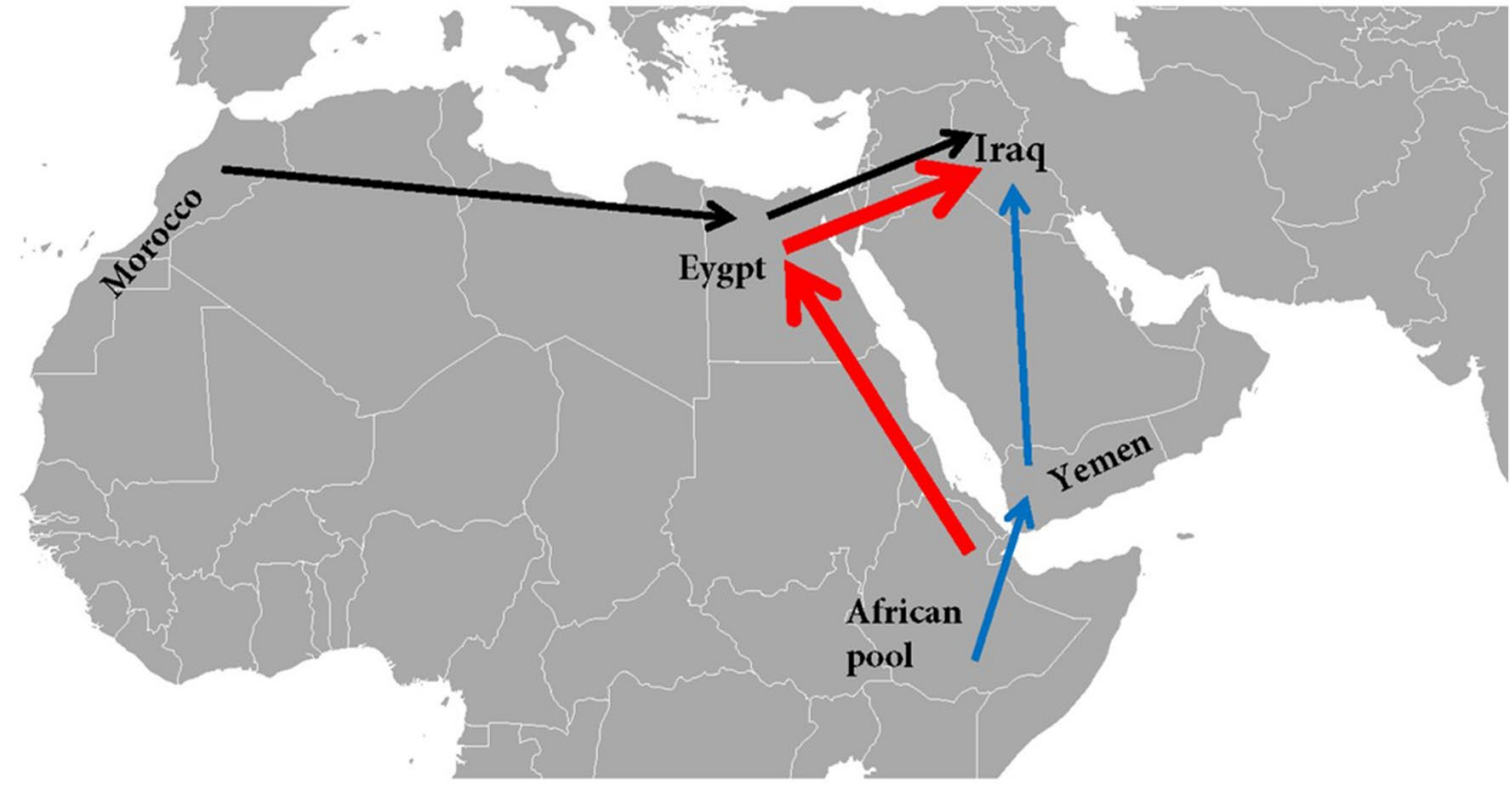
Level one migration routes: Morocco → Egypt → Iraq, Africa → Egypt → Iraq and Africa → Yemen → Iraq. The African populations were represented by one pool formed by four populations: Eritrean, Ethiopian, Djiboutian and Kenyan. The most probable migration route is represented by the red arrows. This figure was prepared by the author using Microsoft Word 2016.

The gene flow was studied at three levels. At level one, the out-of-Africa migration to the Arabian Peninsula, three routes were investigated: Morocco → Egypt → Iraq; Africa → Egypt → Iraq; and Africa → Yemen → Iraq. Published data were used to design the migration models: Moroccan^21^, Egyptian^18^ and Yemeni (YHRD accession number YA003764). The African pool comprised populations from Eritrea, Ethiopia, Djibouti and Kenya^17,19^. Figure 1 shows the three level one out-of-Africa migration routes.

The Y chromosome migration pattern analysis showed that the best model was model 2 (the divergence model) for the route Africa → Egypt → Iraq; it has the highest log marginal likelihood (− 4,341.57), Bayes factor (0) and a probability of 1. The results are shown in Table 2. The least likely route was Africa → Yemen → Iraq in all three models.

**Table 2:** Level one: Y-STR tested models for three routes.

Routes	Models	Log(mL)	LBF	Model-probability
Africa → Yemen → Iraq	1	− 5,889.6	− 1548.03	0
Africa → Yemen → Iraq	3	− 5,862.83	− 1521.26	0
Africa → Yemen → Iraq	2	− 5,186.27	− 844.7	0
Morocco → Egypt → Iraq	1	− 5,077.54	− 735.97	0
Morocco → Egypt → Iraq	3	− 5,016.77	− 675.2	0
Africa → Egypt → Iraq	3	− 4,589.83	− 248.26	0
Africa → Egypt → Iraq	1	− 4,573.96	− 232.39	0
Morocco → Egypt → Iraq	2	− 4,502.85	− 161.28	0
Africa → Egypt → Iraq	2	− 4,341.57	0	1

The three migration routes are Morocco → Egypt → Iraq, Africa → Egypt → Iraq and Africa → Yemen → Iraq. The number in column 2 is the migration model number. The African populations were represented by one pool formed by four populations: Eritrean, Ethiopian, Djiboutian and Kenyan. The order of the models in each route was according to log marginal likelihood and the Bayes factor, the lowest to the highest. *Log(mL)* log marginal likelihood, *LBF* Bayes factor. The least probable route was the route Africa → Yemen → Iraq in all its models (1,2,3).

**Figure 2:**
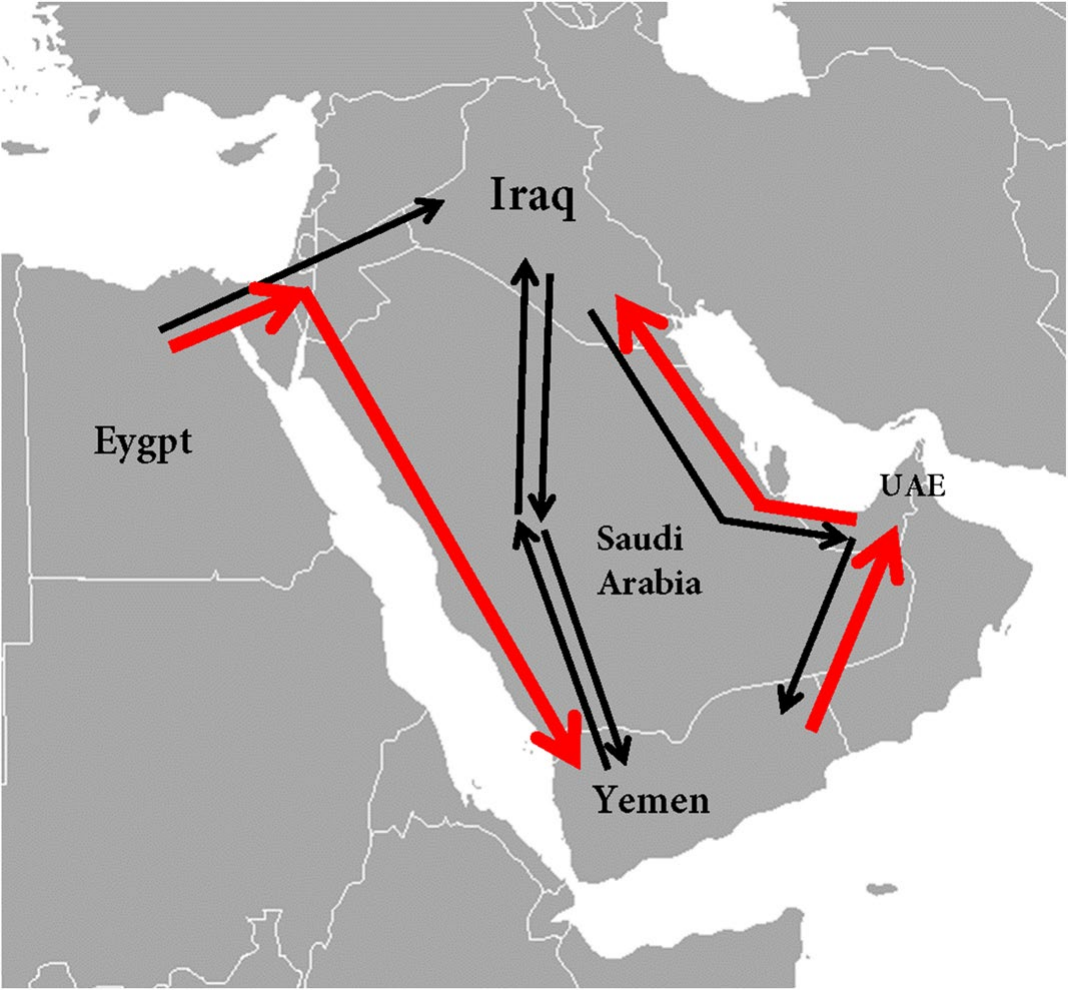
Level two migration routes: gene flow from Egypt across the Sinai Peninsula, to the east towards Iraq and to the south towards Yemen. Four migration routes were tested from Yemen to Iraq, two from Yemen to Iraq, through Saudi Arabia and vice versa, and two from Yemen to Iraq through Emirate and vice versa. The most probable migration routes represented by the red arrows. This figure was prepared by the author using Microsoft Word 2016.

Level two examined population movements inside the Arabian Peninsula. Four routes were investigated, two from Yemen to Iraq, through Saudi Arabia and vice versa, and two from Yemen to Iraq through the UAE and vice versa. The most probable migration route was from Yemen to Iraq through the UAE (model 2) which shows the highest log marginal likelihood (− 5,618.94), Bayes factor (0) and probability of 1. The least probable route was from Yemen to Iraq, models 1 and 3. Level two results are shown in Table 3 and Fig. 2.

**Table 3:** Level two: population movements inside the Arabian Peninsula.

Routes	Models	Log(mL)	LBF	Model-probability
Yemen → Saudi → Iraq	1	− 6,269.71	− 796.76	0
Yemen → Saudi → Iraq	3	− 6,212.4	− 739.45	0
Iraq → Saudi → Yemen	1	− 6,189.77	− 716.82	0
Iraq → Saudi → Yemen	3	− 6,163.98	− 691.03	0
Iraq → UAE → Yemen	1	− 6,046.73	− 573.78	0
Iraq → UAE → Yemen	3	− 6,016.53	− 543.58	0
Yemen → UAE → Iraq	1	− 5,981.27	− 508.32	0
Yemen → UAE → Iraq	3	− 5,957.18	− 484.23	0
Iraq → Saudi → Yemen	2	− 5,781.17	− 308.22	0
Iraq → UAE → Yemen	2	− 5,655.68	− 182.73	0
Yemen → Saudi → Iraq	2	− 5,618.94	− 145.99	0
Yemen → UAE → Iraq	2	− 5,472.95	0	1

Four routes were investigated: Yemen → Saudi → Iraq, Yemen → UAE → Iraq, Iraq → Saudi → Yemen and Iraq → UAE → Yemen. The order of the models in each route was according to log marginal likelihood and the Bayes factor, the lowest to the highest.

*Log(mL)* log marginal likelihood, *LBF* Bayes factor.

The gene flow from Egypt across the Sinai Peninsula was examined in two directions, to the east towards Iraq and to the south towards Yemen. The results show that the most probable route was from Egypt to Yemen with the highest log marginal likelihood (− 3,398.33), Bayes factor (0) and probability of 1 (Table 4, Fig. 2).

**Table 4:** The gene flow from Egypt through the Sinai Peninsula to Iraq and Yemen.

Routes	Models	Log(mL)	LBF	Model-probability
Egypt → Iraq	**2**	− 3,797.3	− 398.97	0
Egypt → Yemen	**2**	− 3,398.33	0	1

The order of the models in each route was according to log marginal likelihood and the Bayes factor, the lowest to the highest.

*Log(mL)* log marginal likelihood, *LBF* Bayes factor.

**Figure 3:**
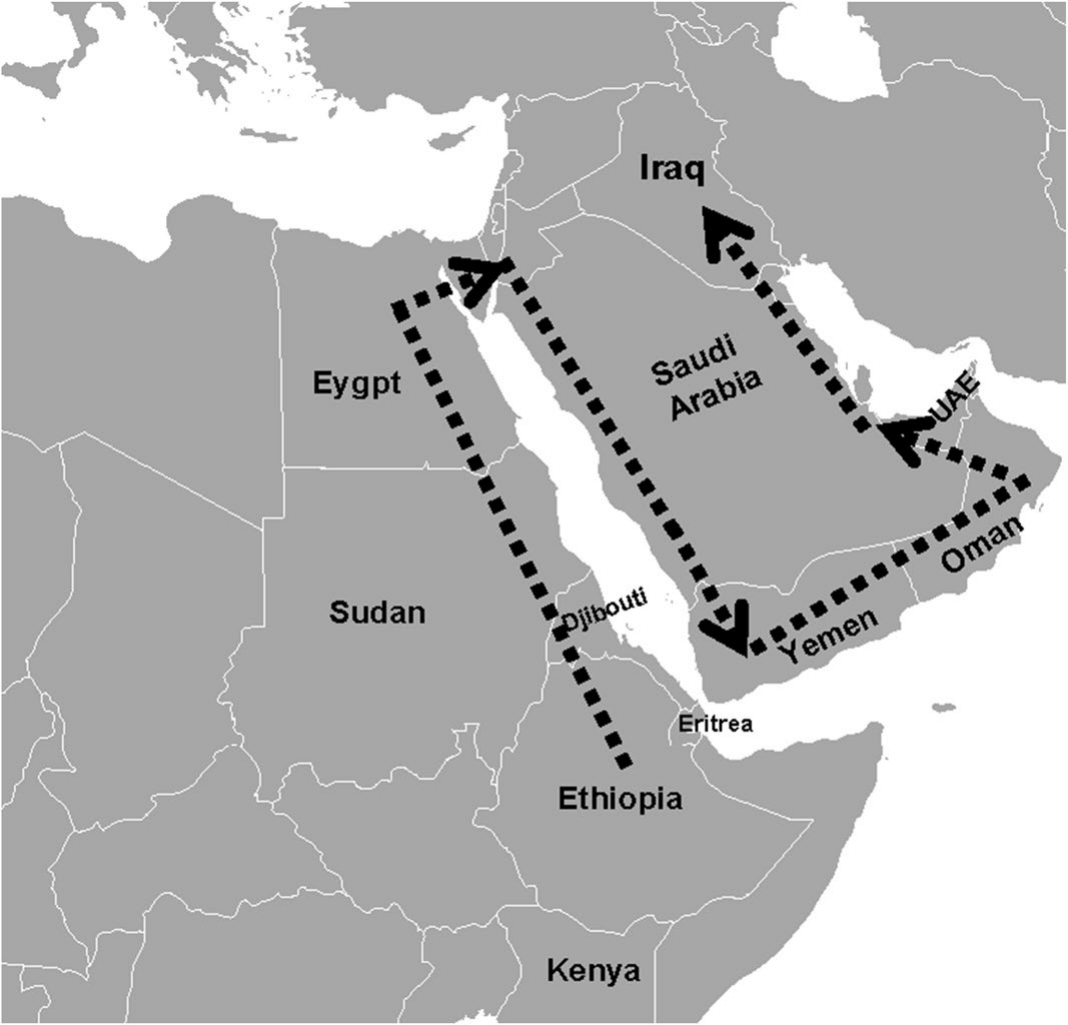
Out-of-Africa gene flow combined with the gene flow inside the Arabian Peninsula. From East Africa to Egypt then around the coast of the Arabian Peninsula to the south reaching Yemen and then to the north through Oman and UAE to reach Iraq. This figure was prepared by the author using Microsoft Word 2016.

The final picture combining the outcomes of levels one and two and according to the most probable routes show that the gene flow to Iraq began from East Africa to Egypt then around the Arabian Peninsula to the south reaching Yemen, and then to the north through the UAE before reaching Iraq. Figure 3 shows the final picture of gene flow from Africa to Iraq. This final picture supports and agrees with the findings of other studies which proposed that the Levantine corridor is the most probable passageway out of Africa^28–30^.

The level three gene flow examined the effect of Iraq and Saudi Arabia on Kuwait. All four migration models in Supplementary Fig. S10 were applied. We found that model 2 dominates this level with the Saudi population having slightly more influence, log marginal likelihood (− 4,536.15), Bayes factor (0) and probability of 1, than the Iraqis on the Kuwaiti population, log marginal likelihood (− 4,701.61). The fourth model which assumed that two populations belong to the same panmictic population is the least probable, indicating that each of the three populations has its own genetic identity. Level three results are shown in Table 5.

**Table 5:** Level three: population movements Iraq → Kuwait, Kuwait → Iraq, Saudi Arabia → Kuwait, and Kuwait → Saudi Arabia.

Routes	Models	Log(mL)	LBF	Model-probability
Iraq + Kuwait	4	− 5,089.68	− 553.53	0
Saudi + Kuwait	4	− 5,061.82	− 525.67	0
Iraq → Kuwait	1	− 5,019.41	− 483.26	0
Saudi → Kuwait	1	− 4,983.58	− 447.43	0
Saudi → Kuwait	3	− 4,944.75	− 408.6	0
Iraq → Kuwait	3	− 4,944.52	− 408.37	0
Iraq → Kuwait	2	− 4,701.61	− 165.46	0
Saudi → Kuwait	2	− 4,536.15	0	1

The order of the models in each route was according to log marginal likelihood and the Bayes factor, the lowest to the highest.

*Log(mL)* log marginal likelihood, *LBF* Bayes factor.

## Discussion

The inclusion of a larger number of Y-STR loci such as those included in the PowerPlex Y-23 kit^31^ was intended to increase the discriminative power and therefore it is a popular kit in forensic casework and population studies. Y-STR haplotypes comprising the Y STRs included in the PowerPlex Y-23 kit were evaluated for their diversity in Iraqi Arab population.

Each population has its own unique genetic structure that can be characterised by its Y-STR haplotype databases for studying variation within, and between, population groups. Such databases are of great value in ascertaining the forensic value of Y-STR evidence. This study shows that the Iraqi Arab population has its own distinctive characteristics which differ from other populations^17^. The comparison of the databases revealed that two loci (DYS389I and DYS392) were less variable in the Iraqi population than in the other populations. Another characteristic feature of the Iraqi database was that the highest genetic diversities were for the dual marker DYS385a/b and a single-locus marker DYS458 at 0.93 and 0.85 respectively, unlike the other populations which showed the highest genetic diversities for the markers DYS385a/b and DYS481^17^.

Four of the six newly introduced markers, namely DYS481, DYS570, DYS576 and DYS643, ranked near the top in terms of genetic diversity, with GD values exceeding 0.70. This observation was consistent with a published global study^17^. PowerPlex Y-23 with its 23 loci proved to be more forensically informative and discriminating for the Iraqi population than the Y-Filer kit, which contained fewer loci.

It is notable that, the high incidence of microvariant alleles, in particular as reported at DYS458 (34.6%), is characteristic of the Middle Eastern populations. Microvariant alleles add to the discriminatory power and the evidential value of a DNA profile, and can further aid in determining haplogroups. We noticed that 98.8% of the Y-chromosomes carrying these DYS458 microvariants were located within haplogroup J1. This agrees with another study^32^ that showed this microvariant allele to overlap with the M267 marker; this has arisen as result of a combination of drift and founder effects, followed by rapid population expansion, in North Africa and the Middle East during human evolution.

In this study we noted two null alleles at the locus DYS576; both samples belonged to haplogroup J2. DYS576 has been reported^17^ as having the second-highest level of null alleles following DYS448 in an Asian population: 28% of the total reported null allele cases. The YHRD (release 62) contained a total of 31 null allele observations in the locus DYS576 out of a total 126,443 haplotypes (0.024%).

In this study, the duplication of 15, 16 at locus DYS19 was observed in three individuals (1.16%). In the YHRD (release 62) this duplication was at a frequency of 0.053%. Many studies have reviewed and addressed such duplications^10,33^ and it is thought to be because the duplicated region, mutating at a rate of approximately 10^−3^ times per generation in a single-step fashion, gives rise to a new allele usually different from the original by a single repeated unit^34^. The three haplotypes that show duplicated alleles 15, 16 were predicted to belong to haplogroup G2a^35^.

Y-haplogroups were inferred through using Whit Athey’s Haplogroup Predictor; the results showed that the most common haplogroup (34.6%) in Iraqi Arabs was J1 as detected earlier^6,36^. Haplogroup J1 (M267) is one of two major sub-haplogroups from the major haplogroup J (M304) found among modern West Asian, North African, Horn of Africa, Southern European, Central Asian and South Asian populations, essentially delineating the Middle East and associated with speakers of Semitic languages, especially Arabic^37,38^. The frequency of the J1 haplogroup is directly proportional to aridity in the Middle East and it increases toward the periphery of the Arabian Peninsula^39^.

A comparison of the accuracy of three haplogroup prediction software packages found that the precision was 98.80% in Whit Athey’s Haplogroup Predictor, 98.19% in Y Predictor by Vadim Urasin 1.5.0, and 97.59% in Jim Cullen’s Haplogroup Predictor^40^. Furthermore, Whit Athey’s Haplogroup Predictor and the median-joining tree complement each other.

The global Y-STR HapMap generated in this study not only showed a stronger geographical proximity of the population samples, but also a stronger sub-grouping of the corresponding populations than the Kidd Ancestry Informative SNPs HapMap, which shows overlapping genotypes of some regions of the world. This can be explained by STRUCTURE handling autosomal markers differently from the haploid markers, since in autosomal analysis STRUCTURE will define clusters by finding Mendelian populations of individuals. Another factor could be the number of individuals in each input population, with more in the Y-STR than the SNPs analysis^27^. Increasing the number of the Kidd Ancestry Informative SNP markers might improve its HapMap discriminatory power between the overlapping populations.

Out-of-Africa migration and peopling of the Middle East has been studied extensively and various routes of migration have been suggested^28–30^.

The Bayesian inference and the coalescence theory in Migrate-n indicated that most of the gene flow of the Y-STR from Africa to Arabia occurred following coastal pathways and crossing the Sinai Peninsula to Arabia. All the migration routes favoured divergence from ancestral populations without an ongoing migration model (model 2) and showed a probability of 1.0.

Two dispersal routes might explain the out-of-Africa model: a northern route through the Sinai Peninsula and the Levant, and a southern route followed the coast around Arabian Peninsula^41–43^.

The southern coastal route crossing the Bab al Mandab Strait (the narrowest point between Africa and Yemen) to Arabia was proposed as an alternative to the northern route in Ice Age because aridity in the Levant was a strong barrier to human expansion^44,45^. It is also thought that modern humans preferred the southern route because the Bab al Mandab Strait was narrow and shallow at that time; there is no geographical evidence of the existence of an intercontinental bridge 80,000 years ago, when such human intercontinental migrations occurred^44,45^. This study shows that this migration route is the less probable one.

This study supports the theory that the Levantine corridor served as a migratory route from East Africa through ancient Egypt into Iraq^46^.

## Material and methods

### DNA sampling

Blood samples were collected with informed consent from 254 Iraqi males in the Paternity Department of the Medico-Legal Institute in Baghdad using FTA cards. A small disc of 1.2 mm diameter was manually punched out of the card, using a Harris Punch, and used for direct amplification of DNA. Ethical permission for recruitment and analysis was provided by the University of Central Lancashire STEMH Ethics Committee (STEMH 246/June 2014). All methods were performed in accordance with the relevant guidelines and regulations.

### DNA amplification

The PowerPlex Y23 System contains 23 loci: DYS576, DYS389I, DYS448, DYS389II, DYS19, DYS391, DYS481, DYS549, DYS533, DYS438, DYS437, DYS570, DYS635, DYS390, DYS439, DYS392, DYS643, DYS393, DYS458, DYS385a/b, DYS456 and Y-GATA-H4. PCRs were conducted using one third of the recommended quantities and a total reaction volume of 8 μl. Amplification was performed using the manufacturer’s recommended cycling conditions. Fragments were detected using an ABI3500 Genetic Analyzer (Thermo Fisher Scientific) using the manufacturer’s recommended protocols. GeneMapper IDX software V1.4 was used for allele calling and interpretation.

### Forensic and population genetic parameters

The haplotype frequencies were calculated by the counting method. Haplotype diversity was estimated by Nei’s formula^47^, HD = (1 − Σ pi2) *n/(n − 1) where n is the sample size and pi is the ith’s haplotype frequency. Genetic diversity (GD) was calculated as 1 − Σ pi2, where pi is the allele frequency. The match probability (MP) was calculated as Σ pi2, where pi is the frequency of the ith haplotype. Discriminatory capacity (DC) was calculated by dividing the number of different haplotypes by the total number of samples in a given population; in the formula DC = h/n, h is the number of different haplotypes in the observed population and n is the total number of the population^48^. The haplotype match probability (HMP) was calculated as HMP = 1 − HD^49^.

Molecular data were obtained for the Iraqi population using Y-STRs based on the PowerPlex Y 23 System, and subjected to comparative analyses with available data on other close and distant populations. Comparison with other datasets required reduction of the number of STRs to a shared set of 15, so that more Middle Eastern populations could be included in this analysis. Arlequin 3.5.2.2 software^23^ was used to calculate the average pairwise differences between (PiXY) and within populations (PiX), in addition to the corrected average pairwise difference between populations (PiXY − (PiX + PiY)/2).

Aiming to assess genetic affinity and structuring of the Iraqi sample, AMOVA computations were performed, considering other populations according to their geographical location; Middle Eastern populations were represented by Yemen, Turkey, Kuwait, Saudi Arabia, Iraq (Kurd), UAE, Qatar and Lebanon; African populations by Morocco, Egypt, Eretria, Ethiopia, Djibouti and Kenya, European populations by Germany, Belgium, Finland and Sweden; and East Asian populations by India, China, Japan and South Korea.

### Iraqi Y haplogroup assignment

The full Y23 haplotypes were used to allocate haplotypes to their most likely haplogroup using Athey’s Haplogroup Predictor^11,12^. DYS549, DYS543 and DYS533 were excluded from the data because the first was not included in the program and the last two because no allele frequency data was available^12^.

The microvariant alleles were truncated to the next lowest integer value since values in the database were treated similarly. Null alleles were simply treated the same as untested markers (T.W. Athey, personal communication).

At GATA-H4, one unit was subtracted from each H4 value to put it on the same basis in the program. There were a number of samples for which the program did not make a prediction (no haplogroup met the criteria), and in those cases the haplotypes were manually examined, with results for some of them (T.W. Athey, personal communication).

### Network analysis on Y chromosome haplogroups

Median-joining networks were constructed using the software NETWORK v5.0.1.0.^26^ and NETWORK Publisher v2.1.1.2 (Fluxus Technology Ltd)^25^. Following the recommendations of the Network’s authors, the intermediate alleles were rounded to the nearest integer; the locus DYS385a/b was removed for network construction. Missing alleles were coded ‘99′ in input files.

### Structure statistical analyses

Population structure was investigated using the program STRUCTURE version 2.3.7^50^ with an admixture model. The HapMap was generated for two panels, the 55 Kidd Ancestry Informative SNPs (AISNPs) genotypes of 140 populations (8,148 individuals)^27^ and Y-STR data for 19 markers of 134 populations (21,323 individuals)^14–17^. Four markers were excluded from the PowerPlex Y23 System, the two rapidly mutating STR (DYS570, DYS576), and the markers (DYS549, DYS643), so that more Middle Eastern populations could be included in this analysis.

For each run, the number of clusters, K, was specified in advance and values in the range 6–11 was used for both Y-STR data and the Kidd AISNPs data. For both tests the program was run with 10,000 burn-ins and 10,000 Markov Chain Monte Carlo (MCMC) iterations.

To assess and visualise likelihood values across multiple values of K and to detect the number of genetic groups that best fit the data, STRUCTURE output was processed with STRUCTURE HARVESTER^51^. Then the multiple replicate analyses of each data set were aligned using CLUMPP^52^ and the output files were used to draw the two HapMaps using Distruct^53^.

### Estimation of migration rate in Iraqi population

Migration rates between other populations and Iraqi were inferred with the MIGRATE program version 4.2.14^54^ using coalescence theory.

The Bayesian inference procedure was chosen for the estimation of population genetic parameters. One long chain was run, with a long sampling increment of 1,000. The sampling increment allows a wider search of genealogy space since not every genealogy will be sampled. The number of discarded trees per chain (burn-in) was set to 5,000. According to the increment value and the number of discarded trees, each sample was visited 5,000,000 times (P. Beerli, personal communication).

Metropolis-Coupled MCMC (“MCMCMC”) or “heating” was applied for auxiliary searches with more permissive acceptance criteria^55–57^. The search was run with four chains at different temperatures (1.0, 1.5, 3.0, and 10,000) with an adaptive heating scheme that manipulated the temperatures according to their swapping success (P. Beerli, personal communication). The hotter chains move more freely and explore more genealogy space than the cold chains.

Input data files were prepared using the PGD Spider data converting tool^58^. Gene flow was investigated at three levels: level one is the out-of-Africa migration to the Arabian Peninsula; level two investigated the movement of Arabs inside the Arabian Peninsula; and level three investigated the migration rate between the three neighbouring countries Iraq, Saudi Arabia and Kuwait.

Four gene flow models were designed. The first model represents direct migration from one population to the other, the second divergence from an ancestral population and the third divergence from the ancestral population with ongoing immigration. The fourth model assumes that two populations belong to the same panmictic population, and is only used in level three. The log marginal likelihood of the different runs was used to generate the Bayes factors. The Bayes factors were used for model comparison, where their magnitudes give evidence of how different the models are. Supplementary Figure S10 shows the migration models that were used in this study.

## Supplementary information

Supplementary Legends.

Supplementary Figure S1.

Supplementary Figure S2.

Supplementary Figure S3.

Supplementary Figure S4.

Supplementary Figure S5.

Supplementary Figure S6.

Supplementary Figure S7.

Supplementary Figure S8.

Supplementary Figure S9.

Supplementary Figure S10.

Supplementary Tables.

## Data Availability

The materials, data and associated protocols are available to readers without undue qualification in material transfer agreements.
