# Supplementary material for: Population genetic diversity in an Iraqi population and gene flow across the Arabian Peninsula
Source: Sci Rep. 2020 Sep 17;10:15289. doi: 10.1038/s41598-020-72283-1 (PMC7499422; doi:10.1038/s41598-020-72283-1)
Supplement: Supplementary file 1 — Supplementary Legends. [file 41598_2020_72283_MOESM1_ESM.docx]

**Supplementary Figure S1 Political borders of Iraq and its position in the Middle East.** Iraq is bordered by the Arabian Gulf, Kuwait, Saudi Arabia in the south, Jordan and Syria in the west, Turkey in the north, and Iran in the east ([www.lib.utexas.edu](http://www.lib.utexas.edu)) [5].

**Supplementary Figure S2 The match probability and genetic diversity for each of the 23 loci in the Iraqi population ranked from the highest genetic diversity to the lowest**. DYS389II.I represents the values of DYS389II calculated as the difference between DYS389II and DYS389I. This figure was prepared by the author using Microsoft Excel 2016.

**Supplementary Figure S3 The matrix of pairwise genetic distance R_st_ of Y-STR between the Iraqi population and the other populations.** The Iraqi Arab was closest to the Iraqi (Kurds), the Yemeni and the Kuwaiti populations, and furthest from the Djiboutian, the Ethiopian and the Turkish populations. Comparison with other datasets required reduction of the number of STRs to a shared set of 15. The scale of differences is shown on the right side of the matrix. This matrix was generated using R statistical software version 4.0.1 [24].

**Supplementary Figure S4 Matrix plot showing population average pairwise differences based on 15 loci.** The area above the diagonal (green) shows the average number of pairwise differences between populations (PiXY); the diagonal (orange) shows the average number of pairwise differences within population (PiX); and below the diagonal (blue) shows the corrected average pairwise difference (PiXY-(PiX+PiY)/2). Comparison with other datasets required reduction of the number of STRs to a shared set of 15. The scale of differences is shown on the right side of the matrix. This matrix was generated using R statistical software version 4.0.1 [24].

**Supplementary Figure S5 Dendrogram of the 23 populations based on the Rst values.** Four clusters were created. Iraq (Arab), Iraq (Kurd), Yemen and Kuwait fell into one cluster and the rest of the Middle Eastern populations fell into one cluster. This dendrogram was generated using R statistical software version 4.0.1 [24].

**Supplementary Figure S6 Multidimensional scaling (MDS) plots of Middle Eastern populations based on Rst values.** The MDS figure was generated using the R statistical software version 4.0.1 [24].

**Supplementary Figure S7 Median-joining network for 254 Iraqi Arab haplotypes, constructed from data on 21 Y-STRs.** Circles represent haplotypes, with the area proportional to sample size, and lines between them proportional to the number of mutational steps. The colours representing the haplogroups are indicated in the key, left. UP=unpredicted. The Median-joining network was edited using NETWORK Publisher v2.1.1.2 (Fluxus Technology Ltd) [45].

**Supplementary Figure S8 The HapMap of the Kidd Ancestry Informative SNPs (AISNPs)** **from 140 populations (8,148 individuals) showing 10 clusters.** This HapMap was generated using the Distruct program [53].

**Supplementary Figure S9 The HapMap of the Y-STR haplotypes using 19 STR markers from 134 populations (21,323 individuals) showing 9 clusters.** This HapMap was generated using the Distruct program [53].

**Supplementary Figure S10 A schematic depiction of the migration models that were used to examine the migration rate for Y-STR data for the Iraqi population.** Model 1 represents direct migration from population A to B and then from B to C. In Model 2 population C diverged from A and B diverged from C. Model 3 represents divergence from the ancestral population with ongoing immigration. Model 4 assumed that two populations belong to the same panmictic population; this model is used only in level three. This figure was prepared by the author using Microsoft Word 2016.
