# Supplementary figures and images for: Population genetic diversity in an Iraqi population and gene flow across the Arabian Peninsula
Source: Sci Rep. 2020 Sep 17;10:15289. doi: 10.1038/s41598-020-72283-1 (PMC7499422; doi:10.1038/s41598-020-72283-1)

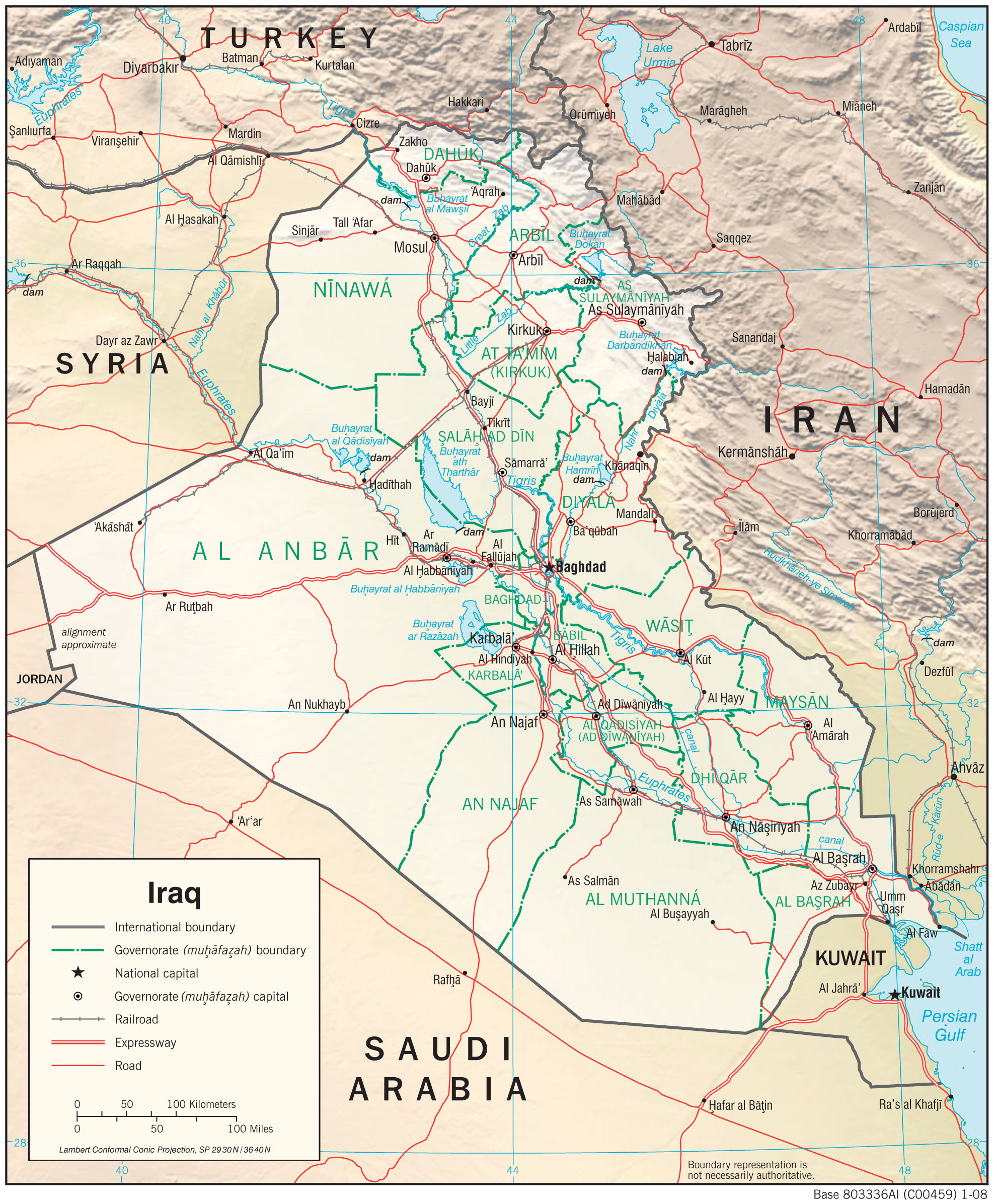

Supplement: Supplementary file 2 — Supplementary Figure S1. [file 41598_2020_72283_MOESM2_ESM.jpg]

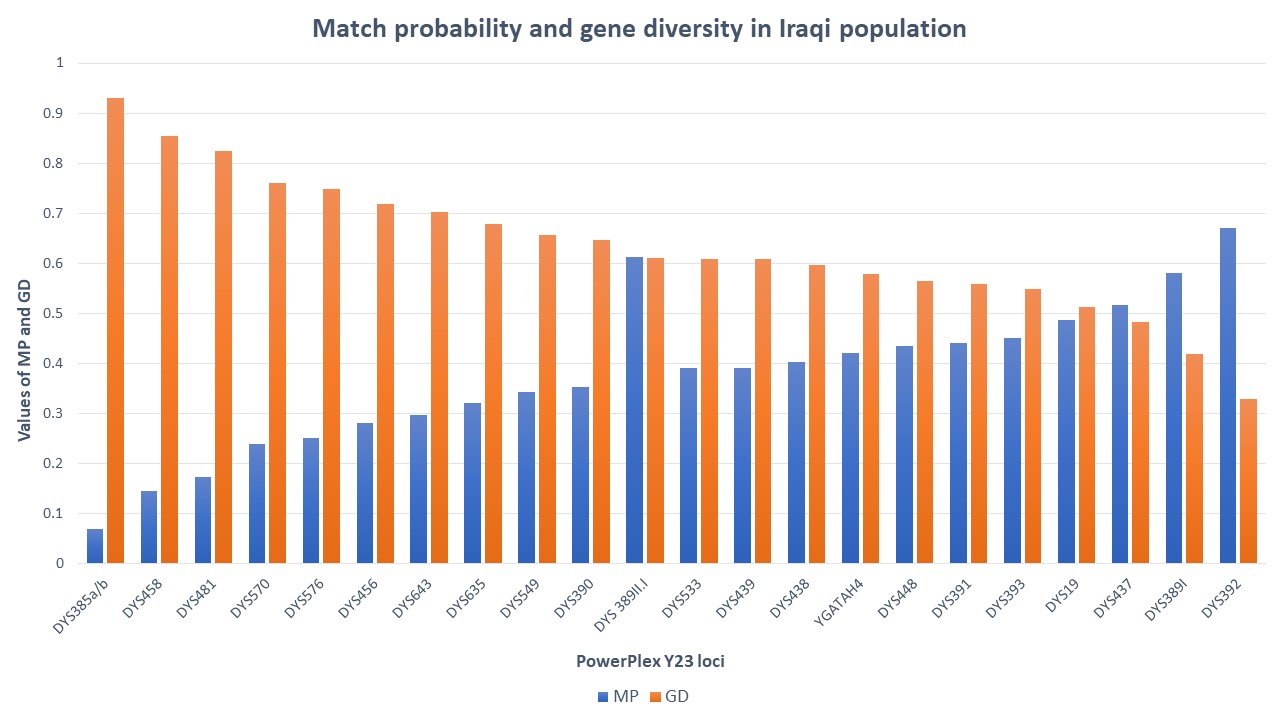

Supplement: Supplementary file 3 — Supplementary Figure S2. [file 41598_2020_72283_MOESM3_ESM.jpg]

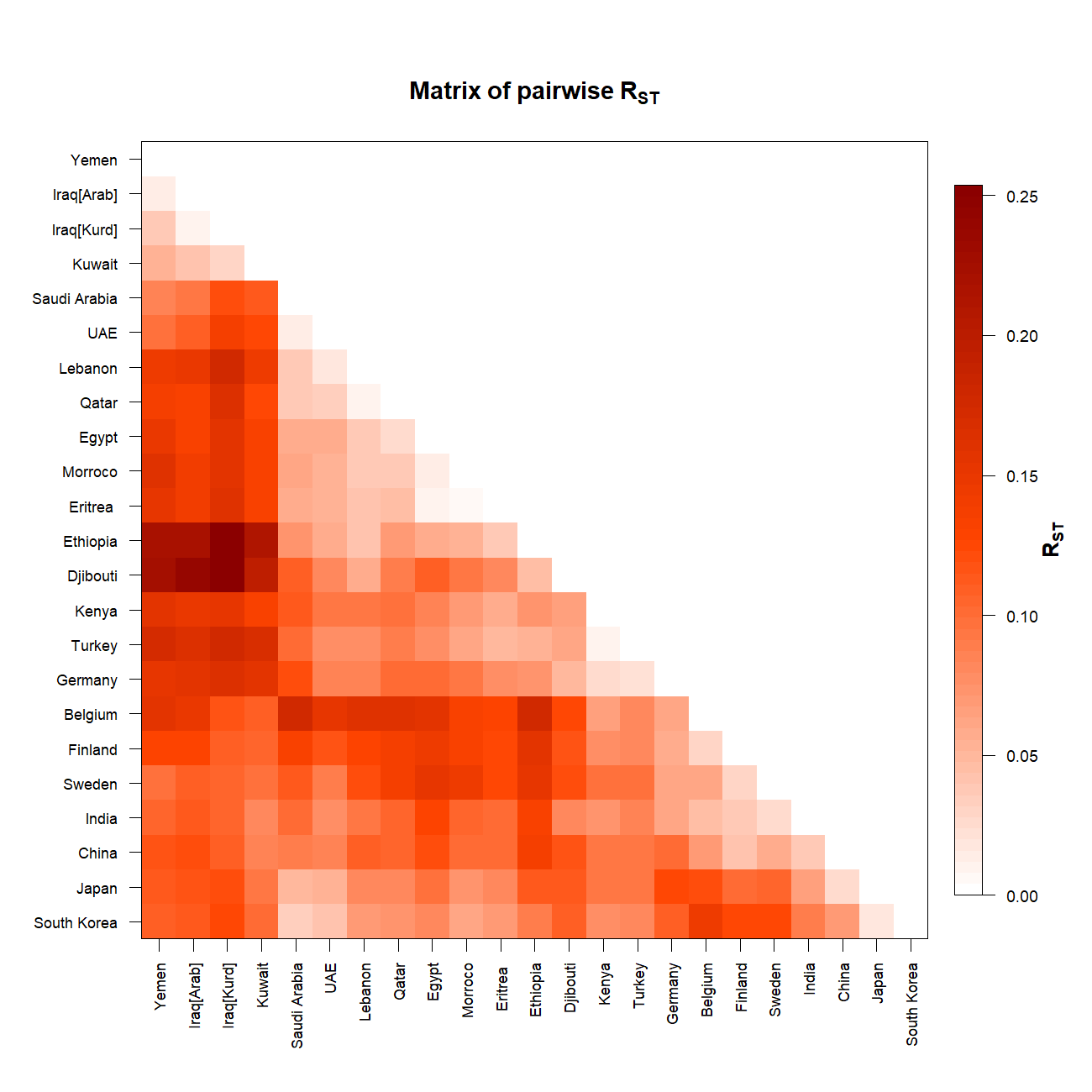

Supplement: Supplementary file 4 — Supplementary Figure S3. [file 41598_2020_72283_MOESM4_ESM.jpg]

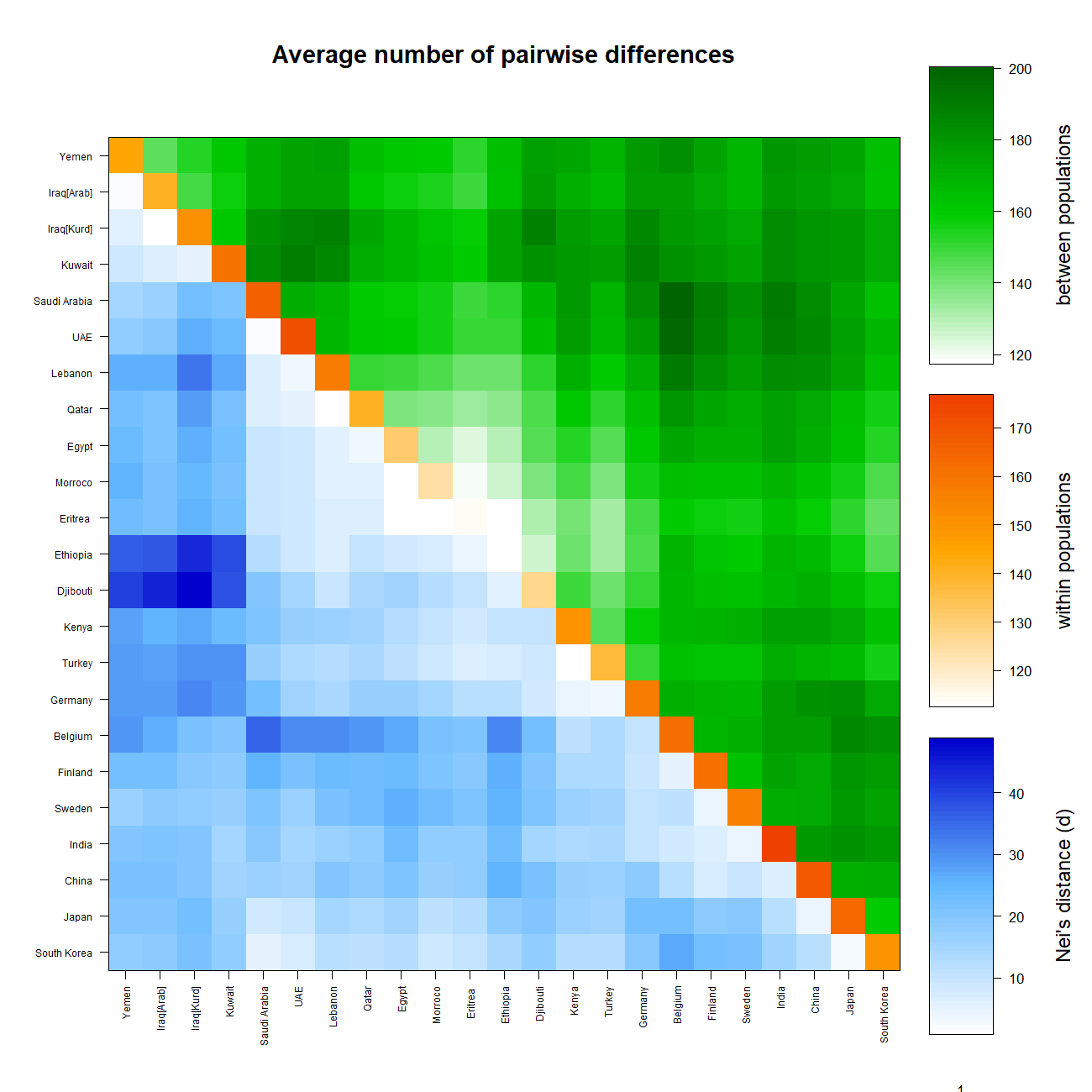

Supplement: Supplementary file 5 — Supplementary Figure S4. [file 41598_2020_72283_MOESM5_ESM.jpg]

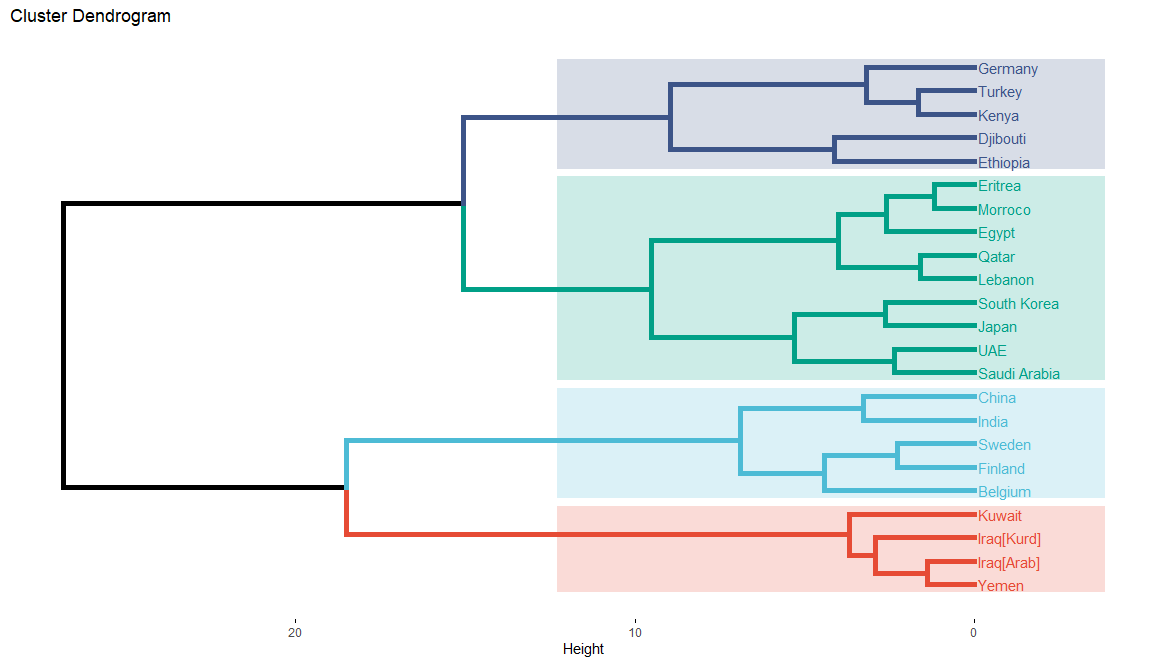

Supplement: Supplementary file 6 — Supplementary Figure S5. [file 41598_2020_72283_MOESM6_ESM.jpg]

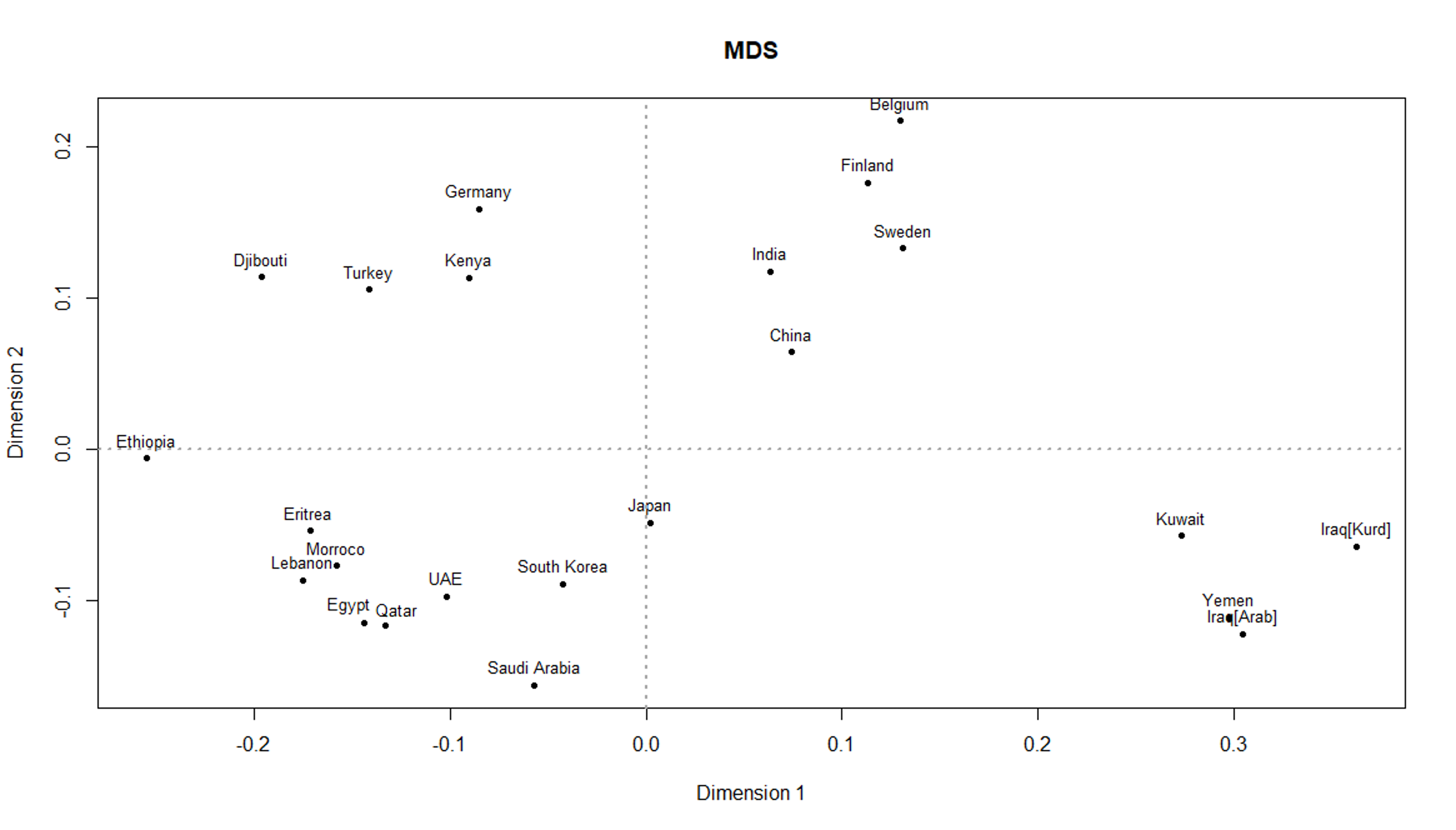

Supplement: Supplementary file 7 — Supplementary Figure S6. [file 41598_2020_72283_MOESM7_ESM.jpg]

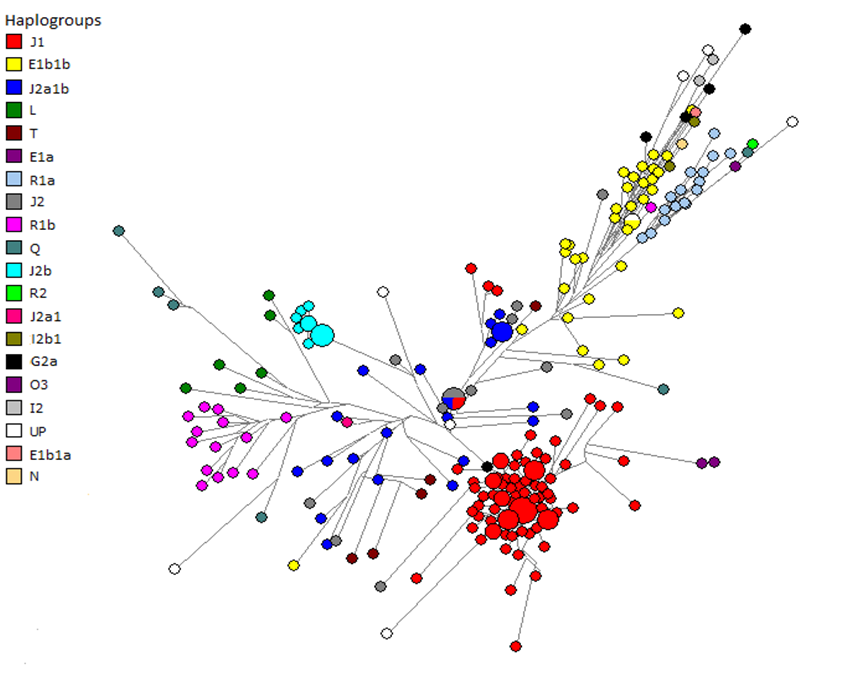

Supplement: Supplementary file 8 — Supplementary Figure S7. [file 41598_2020_72283_MOESM8_ESM.jpg]

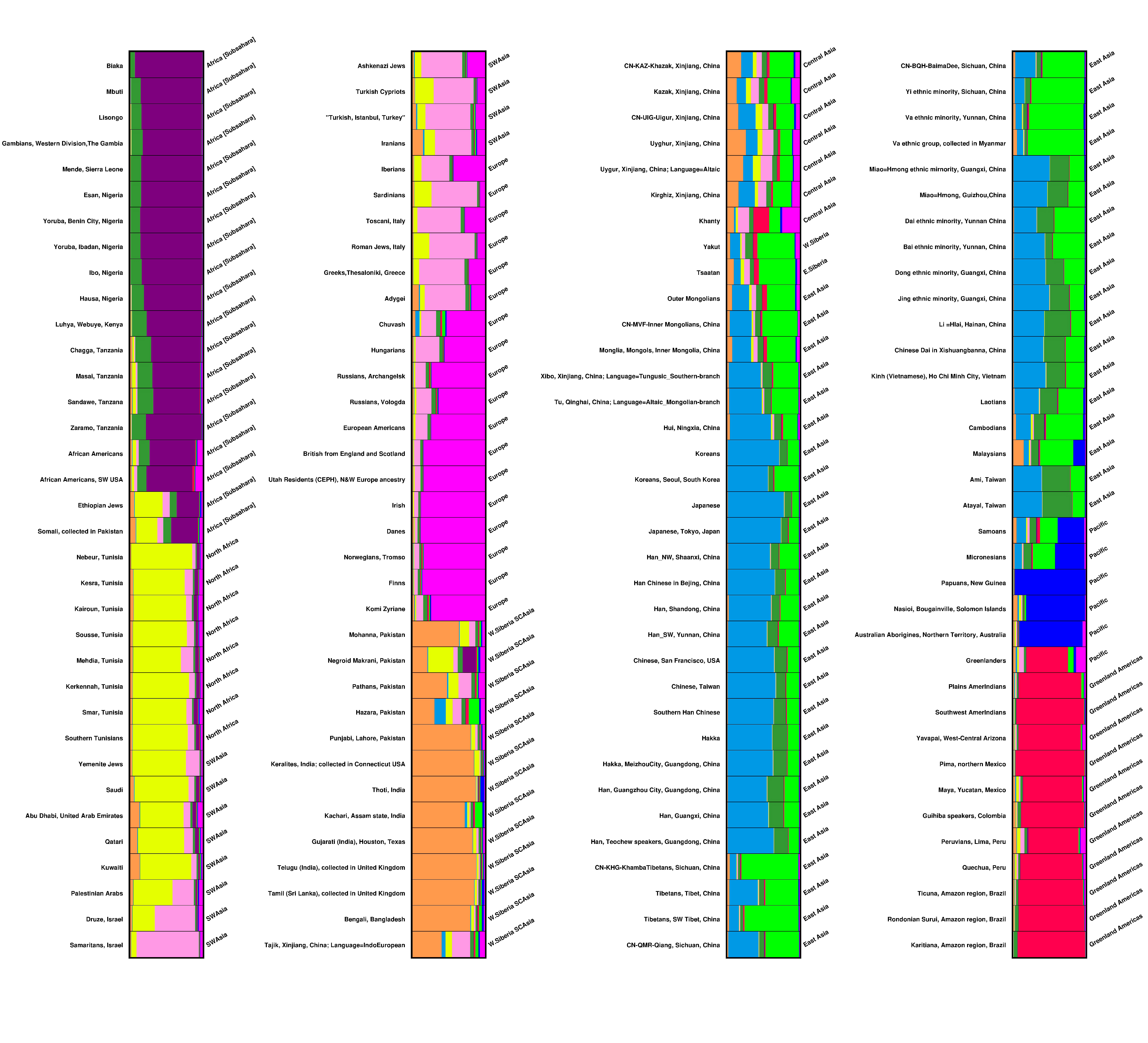

Supplement: Supplementary file 9 — Supplementary Figure S8. [file 41598_2020_72283_MOESM9_ESM.jpg]

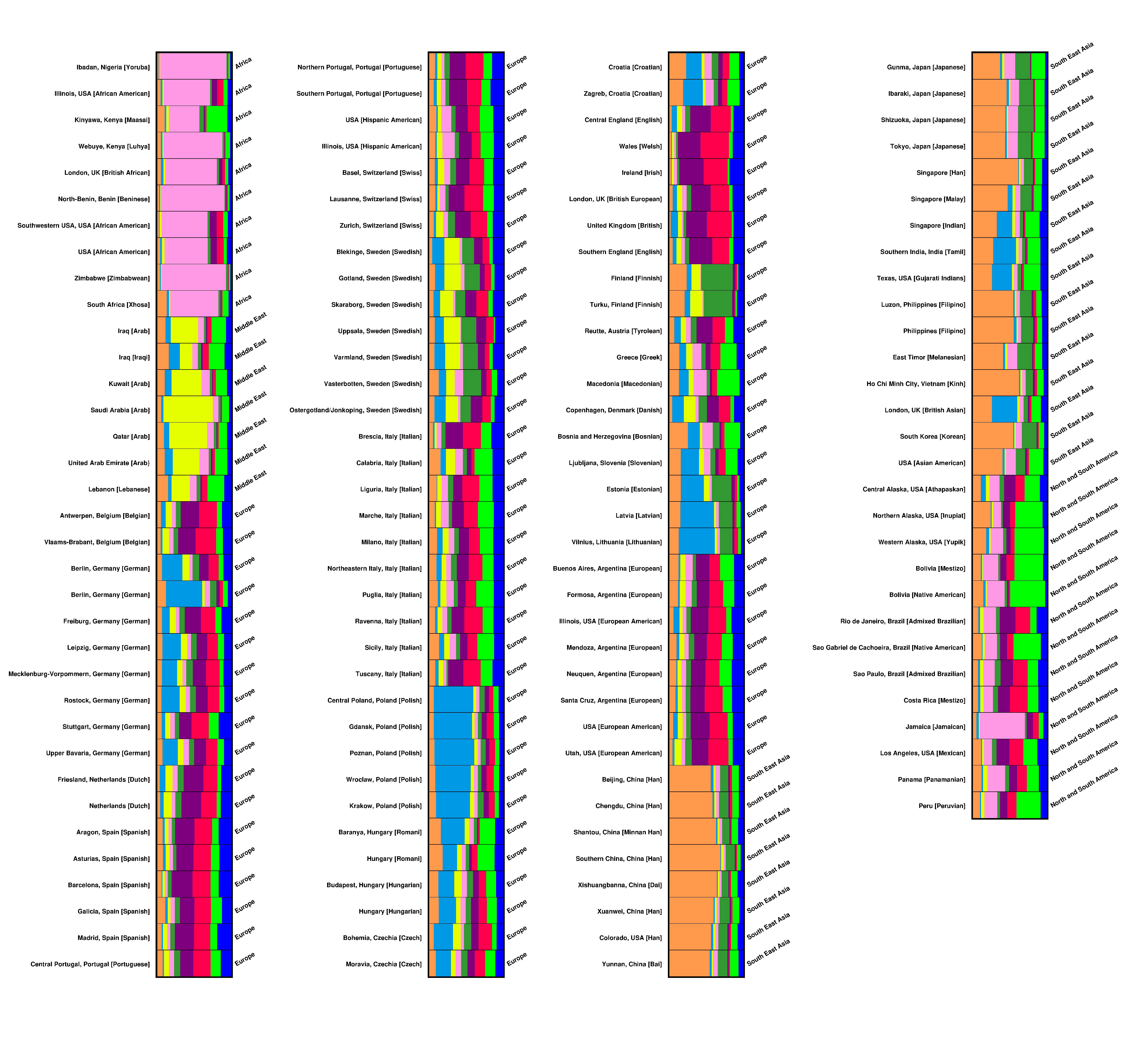

Supplement: Supplementary file 10 — Supplementary Figure S9. [file 41598_2020_72283_MOESM10_ESM.jpg]

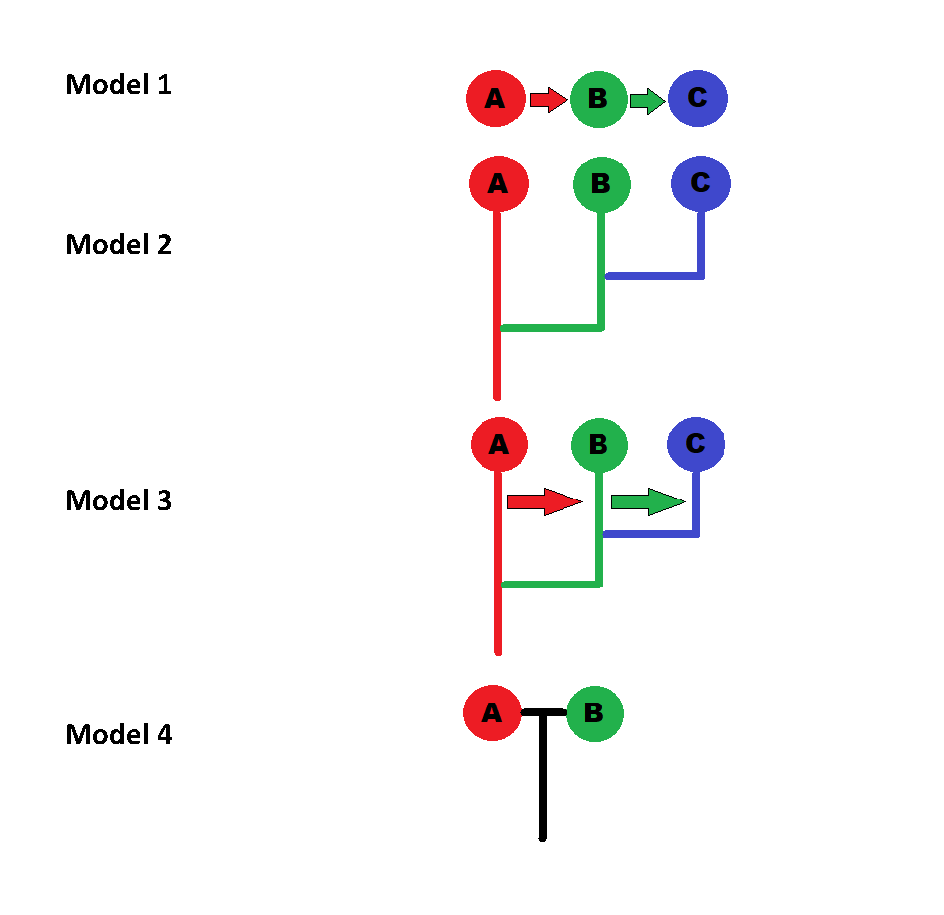

Supplement: Supplementary file 11 — Supplementary Figure S10. [file 41598_2020_72283_MOESM11_ESM.jpg]
